# Heterogeneous Responses to Epidermal Growth Factor Receptor (EGFR) Tyrosine Kinase Inhibitors (TKIs) in Patients with Uncommon EGFR Mutations: New Insights and Future Perspectives in this Complex Clinical Scenario

**DOI:** 10.3390/ijms20061431

**Published:** 2019-03-21

**Authors:** Alessandro Russo, Tindara Franchina, Giuseppina Ricciardi, Alessandra Battaglia, Maria Picciotto, Vincenzo Adamo

**Affiliations:** Medical Oncology Unit A.O. Papardo, Department of Human Pathology, University of Messina, 98158 Messina, Italy; alessandro-russo@alice.it (A.R.); tfranchina@unime.it (T.F.); giusyricciardi81@hotmail.it (G.R.); alessandrabattaglia88@gmail.com (A.B.); mpicciotto@unime.it (M.P.)

**Keywords:** EGFR, afatinib, erlotinib, NSCLC, uncommon mutations, rare mutations, osimertinib, poziotinib, S768I, exon 20 insertions

## Abstract

Uncommon Epidermal Growth Factor Receptor (EGFR) mutations represent a distinct and highly heterogeneous subgroup of Non-Small Cell Lung Cancers (NSCLCs), that accounts for approximately 10% of all EGFR-mutated patients. The incidence of uncommon EGFR mutations is growing, due to the wider adoption of next-generation sequencing (NGS) for diagnostic purposes, which enables the identification of rare variants, usually missed with available commercial kits that only detect a limited number of EGFR mutations. However, the sensitivity of uncommon mutations to first- and second-generation EGFR Tyrosine Kinase Inhibitors (TKIs) is widely heterogeneous and less well known, compared with classic mutations (i.e., exon 19 deletions and exon 21 L858R point mutation), since most of the pivotal studies with EGFR TKIs in the first line, with few exceptions, excluded patients with rare and/or complex variants. Recently, the third generation EGFR TKI osimertinib further revolutionized the therapeutic algorithm of EGFR-mutated NSCLC, but its role in patients harboring EGFR mutations besides exon 19 deletions and/or L858R is largely unknown. Therefore, a better knowledge of the sensitivity of uncommon mutations to currently available EGFR TKIs is critical to guiding treatment decisions in clinical practice. The aim of this paper is to provide a comprehensive overview of the treatment of NSCLC patients harboring uncommon EGFR mutations with currently approved therapies and to discuss the emerging therapeutic opportunities in this peculiar subgroup of patients, including chemo-immunotherapy combinations, next-generation EGFR TKIs, and novel targeted agents.

## 1. Introduction

Treatment of advanced Non-Small Cell Lung Cancer (NSCLC) has considerably improved over the past decade, with the identification of clinically relevant molecular subgroups of patients that may benefit from targeted therapies and Epidermal Growth Factor Receptor (EGFR)-mutated NSCLC as a shining example [1,2]. EGFR mutations target exons 18–21 of the gene, which encodes part of the tyrosine kinase domain of the receptor (encoded by exons 18–24), can be detected in about 15–20% of Caucasian patients with NSCLC, and identify a specific subgroup of patients with specific clinic-pathological features [1,2,3], including female sex, never-/light-smoking status, Asian ethnicity, and adenocarcinoma histology. Since 2009, when the randomized phase III IPASS (Iressa Pan Asian Study) trial demonstrated the superiority of an EGFR tyrosine kinase inhibitor (TKI), gefitinib, compared with standard chemotherapy as first line therapy for patients harboring EGFR mutations [4], several phase III studies have demonstrated the substantial benefit of this strategy in molecularly selected patients, using both reversible (first generation) and irreversible (second generation) EGFR TKIs [1]. Based on the positive results of these trials, treatment of EGFR-mutated NSCLCs with a first- or second-generation EGFR TKI has become the standard of care for upfront therapy of advanced/metastatic disease in this small molecularly selected subgroup of patients, delaying the use of chemotherapy in subsequent treatment lines. Recently, the mutant-selective third-generation EGFR TKI osimertinib further revolutionized the therapeutic landscape of EGFR-mutated NSCLC, demonstrating superiority to first-generation inhibitors in the upfront setting [5].

However, not all EGFR mutations are created equal and, with the exception of classic mutations (i.e., exon 19 deletions and L858R exon 21 point mutations) that account for approximately 90% of cases, the sensitivity of uncommon mutations to currently available EGFR TKIs is widely heterogeneous and less well known, since most of the pivotal studies with EGFR TKIs in the first line, with only a few exceptions [6,7,8] (Table 1), have included patients with classic EGFR mutations.

Therefore, data on the sensitivity of these mutations are scant and come mostly from small retrospective studies, case series, or case reports. Treatment of patients with uncommon mutations is still a highly debated issue, with no firmly established standard of care in the vast majority of these cases.

Patients with uncommon mutations define a small and highly heterogeneous subgroup of patients with EGFR mutations, with different sensitivity to first- and second-generation EGFR TKIs, that accounts for approximately 10% of all EGFR mutations in both Caucasian and Asian patients, and present clinic-pathological features that resemble those of other EGFR mutations [19,20,21,22], although some studies have reported an association with smoking history [23,24]. In addition, old age has been associated in patients harboring uncommon mutations with a poorer performance status but a significantly longer progression-free survival (PFS) after treatment with an EGFR TKI (median PFS: 10.5 vs. 5.5 months, *p =* 0.0320) [25]. These mutations include insertions and/or point mutations in the exon 20 (such as S768I), substitutions in the exon 18 (i.e., G719X, E790K/E790A), complex mutations (for example, S768I + G719X), exon 19 insertions or rare variant deletions, and less common mutations in the exon 21 (such as L861Q). However, some of these uncommon mutations, such as exon 18 G719X or exon 20 S768I, do not have a negligible frequency (approximately 1–2% of all non-squamous NSCLCs), comparable to that of other rare oncogene-addicted NSCLC subgroups, such as RET (rearranged during transfection) or ROS1 (c-ros oncogene 1) rearrangements or BRAF (v-Raf murine sarcoma viral oncogene homolog B) mutations [26,27,28], which are under active clinical development. Moreover, their incidence is growing, due to the wider adoption of next-generation sequencing (NGS) for diagnostic purposes, which enable the identification of rare variants, usually missed by available commercial kits that detect only a limited number of EGFR mutations or with low sensitivity methods, such as direct sequencing. Therefore, a better knowledge of the sensitivity of these rare mutations is crucial to guiding treatment decisions in clinical practice. 

In an era of rapidly evolving research, it is important to critically analyze and summarize the evidence reported so far, in order to show the right way to follow. The aim of this paper is to provide a comprehensive overview of the treatment of NSCLC patients harboring uncommon EGFR mutations with currently approved therapies and to discuss the emerging therapeutic opportunities, including chemo-immunotherapy combinations, next-generation EGFR TKIs, and innovative targeted agents.

## 2. Exon 18 Mutations

Exon 18 mutations collectively account for approximately 3–4% of all EGFR mutations and include point mutations, which, in >80% of cases, involve the codons 719 (G719X and the most common variants, G719A, G719S, and G719C) or 709 (E709X), and more rarely, deletion–insertions [19,29,30]. In contrast with other EGFR mutations, an association with the male sex [18] and smoking history has been reported [19,31], with similar sensitivity to chemotherapy as observed in both EGFR wild type and other EGFR mutants [32].

Patients harboring exon 18 mutations benefit from EGFR TKI as first-line treatment, as opposed to chemotherapy (median PFS 14.6 months vs. 5.8 months), although a high level of heterogeneity may be observed, with proximal exon 18 substitutions showing the highest sensitivity to anti-EGFR blockage [32,33].

Preclinical studies have demonstrated an augmented sensitivity of exon 18 mutations to second-generation irreversible EGFR TKIs (i.e., afatinib and neratinib) in comparison to first- or third-generation inhibitors [30].

G719X is the most frequently observed exon 18 mutation for incidence and the second most frequently observed uncommon mutation, after exon 20 insertions. It may be observed as a single point mutation, although it frequently occurs as a complex mutation [19,21]. Preclinical studies have shown that these mutations are oncogenic and are sensitive to EGFR TKI, although they display different sensitivity profiles to these agents. For instance, G719S is less sensitive to gefitinib than erlotinib [34] and G719A is more sensitive to second-generation EGFR TKIs than first- or third-generation agents [30]. These data are in line with a few reports showing lower overall response rate (ORR) (14–53%) in patients harboring G719X mutations, treated with first-generation EGFR TKIs [12,21,35,36], but high ORRs (75–78%) with afatinib [37] and neratinib [38], comparable to those seen in patients with common mutations (Table 2). 

Moreover, increasing evidence suggests a better outcome among patients harboring complex exon 18 mutations, compared with those with single EGFR exon 18 mutations [19,41,50]. Recently, a potential role for immune checkpoint inhibitor, pembrolizumab, was hypothesized in patients harboring G719X and concomitant strong PD-L1 expression (≥50%) [51], although the small number of cases treated do not allow definitive conclusions.

E709X mutations may occur as single or, in a third of the cases, as complex mutations and account for <0.5% of all EGFR mutations [52]. However, the true incidence of these mutations may be underestimated, since some of the most commonly used and FDA-approved commercial kits, such as EGFR Therascreen® or Cobas® EGFR Mutation Test, do not detect E709X mutations or exon 18 deletion–insertions. In preclinical models, these mutations have been associated with less sensitivity to EGFR TKIs, compared with G719X point mutations. As previously reported, these mutations exhibit differential sensitivity in transfected cells to first-/third-generation EGFR TKIs, having greater IC_90_s (>25-fold) with erlotinib, gefitinib, and osimertinib in cells transfected with E709K than those with exon 19 deletions, but no IC_90_s differences with afatinib or neratinib [30]. The activity of afatinib in this rare subgroup of patients is further confirmed by the data of the Afatinib Compassionate Use Consortium (ACUC) that reported, in TKI-pretreated patients harboring complex E709X mutations, an intriguing time-to-treatment failure exceeding 12 months, with an ORR of 50% and a disease control rate (DCR) of 100% [53]. More rare exon 18 mutations reported in responders to EGFR TKIs include V689M, S720P/F, P699S, N700D, G721A, V740A, and L718P [54]. 

Among exon 18 deletions, delE790_T710insD is the most common and accounts for 0.16% of all EGFR mutations, although its frequency may significantly vary, based on the detection method used. Preclinical studies suggest that they are the least sensitive exon 18 mutations to the different classes of EGFR TKIs, although the 90% inhibitory concentrations (IC_90_s) of erlotinib, afatinib, and dacomitinib are lower than their trough concentrations at the recommended doses of each drug [30]. Clinical data are scant, with only one reported case demonstrating a response to erlotinib [55], and no responses (3PD and 2 SD, with a median PFS of 2.3 months) in a case series including five patients treated with gefitinib [41], though they may better respond to second generation EGFR TKIs, as suggested by selected case reports [30,56]. The clinical sensitivity of other exon 18 deletion–insertions is largely unknown, given their very low incidence and the paucity of cases reported. Moreover, in some cases, deletion–insertions of the exon 18 may coexist with other uncommon EGFR mutations, including the exon 20 T790M mutation [51], adding further complexity to the case.

## 3. Exon 19 Insertions and Uncommon Exon 19 Deletion Variants

Exon 19 deletions are the most common EGFR mutations (approximately 45% of all EGFR mutations), which eliminate the conserved motif LREA (residues 747–750), and include several different variants, although the most frequently observed is the del746_A750 [2,52]. However, this subgroup of EGFR mutations is not homogenously sensitive to the EGFR TKIs, since differential sensitivity has been reported between deletions encompassing the amino acids from codons L747 to E749 (LRE fragment) and non-LRE deletions, with a lower response to EGFR TKIs in the latter molecular subgroup [57]. Some of these uncommon exon 19 deletions are missed by commercially available kits for EGFR testing used in daily practice, which target the most common exon 19 deletions [58,59].

Less frequently, EGFR exon 19 may be associated with other genetic events, including small insertions.

Exon 19 insertions are a relatively uncommon family of EGFR mutations with a reported frequency of <0.5% of all EGFR mutations. These insertions exhibit structural similarities with exon 19 deletions and have been associated with in vitro and in vivo sensitivity to first-/second-generation EGFR TKIs [60], although the ORR may be slightly lower (about 40–50%) than reported with classic mutations [61,62], as seen with other uncommon EGFR mutations. 

Finally, several other rare exon 19 mutations have been occasionally reported, and their sensitivity to first-/second-generation EGFR TKIs is variable, including sensitive (L747F, P733L, K757R, E746G, and V742X) and resistance mutations (D761Y, E746V, and L747S) [49,63]. 

## 4. Exon 20 Insertions and Mutations

After classic mutations, exon 20 insertions are the third most common family of EGFR mutations. These insertions encompass residues from 762 to 775 (spatially located after the C-helix of the EGFR kinase domain) and represent a highly heterogeneous family of EGFR mutations, with over 64 unique variants described to date, with an estimated incidence of up to about 10% of all EGFR mutants in NSCLC [64,65,66,67]. Exon 20 insertions occur in patients with clinic-pathological features that resemble those of classic EGFR mutations. Traditionally, they have been thought to be non-responsive mutations, however their high level of molecular heterogeneity suggests that these mutations may, at least in part, respond to EGFR TKIs, especially those between codons 762–768 [64] or those containing a glycine at position 770 [68]. Structural differences within the exon 20 insertions may be responsible for the highly variable sensitivity of these mutations. Indeed, most of these mutations are TKI-resistant and present, as reported for the D770_N771 insNPG mutation, an altered ATP-binding pocket, adopting an active conformation. Others are TKI-sensitive, for example, the exon 20 A763_Y764 insFQEA (6% of all exon 20 insertions, <1% of all EGFR mutations), which presents a structure and an enzyme kinetic that resembles that of the exon 21 L858R point mutation [66,69], although the benefit of EGFR TKIs may be shorter than that observed with common mutations (overall response rate of 73% and a time to progression of five months) [61]. 

Patients harboring exon 20 insertions are associated with an ORR of 0–11% and a median progression-free survival (PFS) of 2–3 months when treated with first- or second-generation EGFR TKIs [19,21,37,44,45,70], and are associated with a similar overall survival of EGFR wild type patients [71]. These patients may experience a higher overall response rate (ORR) (58–63%) and a longer PFS (six months) with platinum-doublet chemotherapy, as reported in small retrospective studies [70,71]. Therefore, treatment of these patients currently should be platinum-based chemotherapy in the first instance, reserving the use of first-/second-generation EGFR TKIs only in later stages of the disease, since the benefit of these agents is generally poor and transient, although some patients may experience long-term disease control [19,45], especially those harboring complex mutations [21] and those with proximal insertions [43]. 

Some authors have suggested a potential role for the vertical blockage with an EGFR TKI, in combination with a monoclonal antibody anti-EGFR that reported clinical activity in selected cases [72,73], although the toxicity profile may represent an obstacle for the development of a similar strategy. Recently, in vitro studies reported that osimertinib was effective against EGFR TKIs resistant exon 20 insertions, albeit with IC values 10–100-fold higher than classic mutations with or without T790M [74], suggesting that increased doses may be more useful than commonly used doses in this setting. The relatively more favorable therapeutic window of osimertinib allows for the safe use of high dose schedules. A phase II study is currently testing this agent at 160 mg/daily in patients with EGFR exon 20 insertions and previously treated with at least one chemotherapeutic line for advanced disease (NCT03191149) [75]. Osimertinib is also under evaluation at standard doses (80 mg/daily) in an open label phase II study in chemotherapy pre-treated Korean NSCLCs, harboring exon 20 insertions (NCT03414814). 

The EGFR and Human epidermal growth factor receptor 2 (HER2) exon 20-selective TKI poziotinib (NOV120101, HM781-36B) has shown in vitro activity against EGFR exon 20 insertions with an average IC50 value of 1 nM, approximately 100-fold higher than osimertinib and 40-fold than afatinib. Indeed, poziotinib is smaller and has greater flexibility, allowing it to overcome the steric hindrance in the drug-binding pocket of exon 20 insertions, which it tightly binds [76]. Preliminary data of a phase II study in 50 heavily pretreated EGFR exon 20 mutated NSCLCs have been recently reported, showing an intriguing activity in this rare subgroup of patients, with a 43% confirmed ORR and a median PFS of 5.5 months (CI 95%, 5.2-NA) [77]. These data compares favorably with historical controls, reporting only modest activity with first-/second-generation EGFR TKIs or chemotherapy, although the sensitivity of most EGFR exon 20 insertions variants to this agent is largely unknown. A multicenter phase II confirmatory study is ongoing (NCT 03318939). Similarly, TAK-788 (AP32788) is a novel EGFR/HER2 that inhibits in vitro EGFR exon 20 insertion mutants more potently than WT EGFR. The results of a phase I/II study (NCT02716116) confirmed the preclinical activity in 39 patients harboring EGFR exon 20 insertions, with a 39% ORR at doses of 80–160 mg/day and 94% DCR. Four phase II expansion cohorts have been opened and are evaluating TAK-788 in different molecularly defined subgroups, including patients with EGFR exon 20 insertions, with or without CNS metastases [78]. 

TAS6417 is a novel EGFR inhibitor that targets EGFR exon 20 insertion mutations while sparing wild-type EGFR, which recently demonstrated in vitro and in vivo activity against a diverse range of exon 20 insertions, including A763_Y764insFQEA, D770_N771insG, and H773_V774insPH [79]. These preclinical data provide the rational for clinical development of this agent in this molecularly defined subgroup of EGFR mutations.

Finally, the phase II RAIN study (NCT03805841) is evaluating the role of tarloxotinib (TH-4000), a hypoxia-activated prodrug that releases an irreversible pan-HER TKI, in NSCLC patients harboring EGFR or HER2 exon 20 insertions.

Other studies are pursuing different therapeutic strategies against EGFR exon 20 insertions. The heat shock protein 90 (Hsp90) chaperone complex protects cellular proteins from degradation by the ubiquitin–proteasome system and preclinical studies reported that EGFR exon 20 insertions displayed sensitivity to the Hsp90 inhibitor, luminespib (AUY922) [80]. A phase II study in EGFR exon 20 insertions reported a modest activity in this molecularly defined subgroup, with a 17% ORR, a 38% DCR, and a 2.8 month median PFS [81]. Despite these modest results, the unique mechanism of action of this agent suggests a potential role for luminespib in exon 20 insertions, likely in patients with primary or secondary resistance to mutant-selective EGFR TKIs.

Among exon 20 mutations, the substitution S768I is one of the most well characterized. This point mutation occurs in approximately 1–2% of EGFR mutated NSCLCs, demonstrated transforming capacity in vitro, and it has been associated with variable responses to EGFR TKIs, but collectively seems less sensible than common mutations to first generation agents [82,83]. The EGFR S768I point mutation may occur either alone or as a part of a complex mutation. One of the most frequent compound mutations is the G719X + S768I (about 1% of all EGFR mutations) [37,84], which has been shown to be sensitive to the first-generation EGFR TKIs, but to a lesser extent than classic mutations (ORR approximately 50% and median PFS of 8–10 months) [12,33,46]. However, preclinical studies have demonstrated an augmented sensitivity of exon 18 mutations to second-generation irreversible EGFR TKIs (i.e., afatinib and neratinib), in comparison to first- or third-generation inhibitors [30], with a reported ORR of 75–78% in patients with G719X mutations in clinical trials [37,38], comparable to that of patients with common mutations. Similarly, S768I mutations exhibit differential sensitivity to EGFR TKIs, with a higher sensitivity to afatinib in preclinical models compared with first-/third-generation EGFR TKIs [85], with an ORR of 100% and a median PFS of 14.7 months in the combined analysis of the LUX-Lung 2, 3, and 6 trials [37]. Clinical data with first generation EGFR TKIs are conflicting and collectively suggest that this mutation is less sensitive to these agents [63,86] (Table 3). These preclinical and clinical data suggest that double mutant G719X/S768I patients may exhibit increased sensitivity to afatinib and should be considered the reference EGFR TKI in this rare subgroup of patients.

## 5. Uncommon Mutations of Exon 21

Many other point mutations in the exon 21 may occur besides L858R, and include a heterogeneous subgroup of EGFR mutations associated with varied sensitivity to first-/second-generation EGFR TKIs. These uncommon exon 21 mutations are collectively associated with lower sensitivity to the EGFR TKIs, compared with the classic L858R mutation, with a shorter median PFS (4.5 months vs. 10.4 months, *p =* 0.003) and overall survival (OS, 12.2 months vs. 16.9 months, *p =* 0.04) [32]. The second most frequent exon 21 mutation after L858R is the point mutation L861Q that accounts for approximately 1–2% of all EGFR mutations [42,84] and has oncogenic activity similar to the L858R mutation [85]. Preclinical data suggest that this mutation is sensitive to various EGFR TKI, although it is less sensitive to erlotinib (IC_50_ 92–103 nM) or gefitinib (IC_50_ 170 nM), compared with L858R (IC_50_ 4.5–6 nM), but shows more similar sensitivity to afatinib and osimertinib (IC_50_ 0.5 and 9 nM, respectively) than L858R (IC_50_ 0.2 and 2.5 nM, respectively) [34,85]. A possible explanation for the lower sensitivity to reversible EGFR TKIs, compared with irreversible inhibitors, is the high binding affinity of this mutation to the ATP [88]. These preclinical data find further validation in clinical settings, as reported in several studies (Table 4), showing intermediate sensitivity between classical mutations and resistance mutations. Collectively, preclinical and clinical data available to date supports the use of afatinib as the preferable treatment option in patients harboring L861Q mutations, since the activity of other EGFR TKIs is lower (gefitinib/erlotinib) or has not yet been evaluated in a clinical setting (osimertinib).

Other rarer mutations may be identified in the exon 21, but their sensitivity is generally low (L861R, L862V, V851X, A859X) or uncertain (E866K, H825L, P848L, H870Y/R, and G836S) [35,49,54,63], although the limited data available to date do not allow definite conclusions.

## 6. Complex Mutations

The coexistence of multiple EGFR mutations is generally thought to be a relatively rare event, characterized by the contemporary presence of two or more EGFR mutations. However, the true incidence of these genetic events is unknown, with a reported frequency of 4–14% of all EGFR mutations [21,36,46,47].

Patients with compound mutations are associated with improved outcomes among those harboring rare mutations [19,20,48,89], although a high level of heterogeneity exists. Indeed, this subgroup includes patients with co-occurring classic EGFR mutations, co-existence of classic and rare mutations or, finally, multiple rare mutations only. Therefore, the sensitivity of these different classes of compound mutations may significantly vary, as reported in in vitro studies showing a wide spectrum of EGFR TKI sensitivity, including resistant mutations (e.g., T790M/L858R), intermediate sensitive mutations (such as E709A/G719C, Q787R/L858R, and H870R/L858R, E884K/L858R), and activating mutations [90].

These preclinical data are supported also by clinical evidence suggesting that patients with complex mutations containing exon 19 deletions or exon 21 L858R point mutations are associated with a more favorable outcome than those containing rare mutations only [22,43,47,91], with the best objective rates seen in double exon 19 deletions/L858R [45]. Indeed, the efficacy seems to be influenced by the sensitivity of the accompanying mutation, since complex mutations with a coexistence of both common and resistance mutations are generally associated with lower efficacy than usually observed with single common mutation, whereas those containing common mutations and sensitive rare mutations are associated with similar efficacy to single common mutations [90,91]. However, this is not necessarily true for co-occurring exon 20 mutations (excluding T790M), which are generally thought to be resistant mutations, since they have been associated with similar outcomes to other complex mutations involving different exons, as reported in a recent large retrospective analysis (PFS 10 vs. 12 months and OS 27 vs. 31 months, respectively) [33]. De novo T790M mutations constitute a small subgroup of EGFR mutations, which are observed in approximately 3% of all EGFR-mutated NSCLC patients with standard methods [92], and are generally associated with reduced sensitivity to first- or second-generation EGFR TKIs, with an ORR of 14–33% and a median PFS of 1.94–2.9 months [37,45,91]. Therefore, treatment with platinum-based chemotherapy has traditionally been the preferable first-line option in this subgroup of patients. 

Using highly sensitive detection methods, low-level pretreatment T790M mutations can be detected in a significant fraction of patients (up to 65% of cases) and are constantly associated with shorter PFS [93,94,95]. Preclinical studies suggest that dual blockage of EGFR and VEGF is synergic and may be effective in the erlotinib-resistance model [96]. In the phase II study BELIEF, a substantial benefit was noted from the combination of erlotinib plus bevacizumab in the subgroup of patients with pretreatment T790M [97]. The Erlotinib–bevacizumab combination as upfront therapy in EGFR-mutated NSCLCs has been associated with substantial PFS benefit in phase II/III studies over erlotinib therapy alone (16–16.3 months vs. 9.7–12.4 months) [98,99], however, the recent advent of osimertinib in the first line [5], with a more favorable toxicity profile, may represent an obstacle to the clinical implementation of this combination. Moreover, the activity of osimertinib in patients with acquired T790M mutations [100] suggests that this agent may also represent a valuable therapeutic option in patients with pretreatment T790M mutations. The phase II study AZENT (NCT02841579) is currently addressing this question. Different studies are evaluating the potential role of dual blockage EGFR–VEGF, using osimertinib in EGFR-mutated NSCLCs, either as a first- (NCT02803203, NCT02971501) or second-line option (NCT03133546, NCT02789345). 

## 7. Emerging Therapeutic Options: Chemo-Immunotherapy Combinations

The role of immune checkpoint inhibitors (ICIs) has also been explored in EGFR mutated NSCLCs, although data in patients harboring uncommon mutations are lacking. Mounting evidence suggests that NSCLCs harboring EGFR mutations show lower benefit with the use of anti-PD-1/PD-L1 agents, compared with EGFR wild type. A possible explanation for this phenomenon is that EGFR-mutated NSCLCs are associated with an uninflamed phenotype and weak immunogenicity, exhibiting lower PD-L1 expression/CD8+ TILs in the tumor microenvironment [101,102]. In addition, a linear correlation between tumor mutational burden (TMB) and ORRs with anti-PD-1/PD-L1 agents in multiple solid tumors, including NSCLC, has been reported [103], but EGFR-mutated NSCLCs have a lower TMB (median mutational load: 60) compared with other NSCLCs (TP53 mutated: 325; KRAS mutated: 179; STK11 mutated: 132) [104], with no relative differences between canonical exon 19 del/L858R mutations and exon 20 insertions [66]. These data suggest that immune checkpoint inhibitors targeting PD-1/PD-L1 have, to date, a limited role in EGFR-mutated NSCLCs. However, patients with strong PD-L1 expression and smoking history might benefit from immunotherapy [105,106], including those with uncommon mutations, as recently reported in a small case series [51]. Another potential therapeutic approach is the use of immunotherapy in association with chemotherapy, although most of the clinical trials with these combinations excluded oncogene-addicted NSCLCs [107]. The role of ICIs plus chemotherapy combinations in EGFR-mutated NSCLCs is unclear. In the IMpower 150 trial, atezolizumab combined with carboplatin/paclitaxel/bevacizumab was associated with a significant improvement, in terms of OS (median OS N.R. vs. 17.5 months, HR 0.54), compared with the same regimen without the anti-PDL1 agent [108], although these results were not confirmed in the IMpower 130 trial comparing carboplatin/nab-paclitaxel + atezolizumab vs. chemotherapy alone (median OS 14.4 vs. 10.0 months, HR 0.98) [109]. These data suggest that carboplatin/paclitaxel/bevacizumab/atezolizumab may represent a therapeutic option in EGFR-mutated NSCLCs and might be an attractive approach for patients with common mutations after failure of first-line EGFR targeting therapy with first-/second-generation EGFR TKIs and who do not acquire the secondary T790M mutation. It may also be applied after failure of first-line osimertinib therapy and in patients with uncommon EGFR mutations and a known resistance to currently available EGFR TKIs, for whom platinum-based chemotherapy is the standard treatment.

## 8. Conclusions

The therapeutic landscape of EGFR-mutated NSCLC is rapidly evolving, and novel mutant-specific third-generation EGFR TKIs are moving to first-line therapy [110]. The use of these agents may revolutionize the current therapeutic algorithm in EGFR mutants and may extend the use of EGFR inhibition, even in patients harboring mutations with known resistance to first-/second-generation EGFR TKIs. However, the sensitivity of uncommon EGFR mutations to the different classes of EGFR TKIs may significantly vary, since some have increased sensitivity to second-generation EGFR TKIs (i.e., exon 18 mutations and S768I exon 20 point mutation), whereas others are unresponsive to first-/second-generation EGFR TKIs, but sensitive to mutant-selective EGFR inhibitors, such as osimertinib and poziotinib (i.e., exon 20 insertions). The highly heterogeneous nature of these mutations, encompassing the complete spectrum of sensitivity to EGFR TKIs, suggests that each of these rare variants should be analyzed separately in clinical studies and that clinical recommendations should be made on a case-by-case basis.

A deeper knowledge of the molecular structure of these rare genetic events along with prospective registries and clinical studies limited to patients with uncommon EGFR mutations is eagerly anticipated, in order to expand the growing list of exploitable driver mutations in NSCLC.

## Figures and Tables

**Table 1:** Phase IIb/III trials evaluating three generations of Epidermal Growth Factor Receptor (EGFR) tyrosine kinase inhibitors (TKIs) in the first-line setting, either compared with platinum-based chemotherapy or other EGFR TKIs.

Trial Name	Selection Criteria	Treatment Arms	*n*	Patients with Uncommon Mutations (%)	ORR (%)	PFS (mos)	OS (mos)	Ref.
IPASS	East-Asian, former or light smokers, adenocarcinoma	Gefitinib vs. Carboplatin/Paclitaxel	132 * vs. 129 *	8 (1.3) ** vs. 13 (2.2) **	71.2 vs. 47.3	9.6 vs. 6.3	21.6 vs. 21.9	[4,9]
First-SIGNAL	Korean, never-smokers, adenocarcinoma	Gefitinib vs. Cisplatin/Gemcitabine	26 * vs. 16 *	Excluded	84.6 vs. 37.5	8.0 vs. 6.3	27.2 vs. 25.6	[10]
WJTOG 3405	Japanese, EGFR mutated	Gefitinib vs. Cisplatin/Docetaxel	86 vs. 86	Excluded	62.1 vs. 32.1	9.2 vs. 6.3	35.5 vs. 38.8	[11]
NEJ 002	Japanese, EGFR mutation	Gefitinib vs. Carboplatin/Paclitaxel	114 vs. 114	5 (4.4%) vs. 5 (4.4%)	73.7 vs. 30.7	10.8 vs. 5.4	27.7 vs. 26.6	[8,12]
NEJ 002	Japanese, EGFR mutated	Gefitinib vs. Carboplatin/Paclitaxel	114 vs. 114	7 (6.1%) vs. 7 (6.1%)	73.7 vs. 30.7	10.8 vs. 5.4	27.7 vs. 26.6	
OPTIMAL	Chinese, EGFR mutated	Erlotinib vs. Carboplatin/Gemcitabine	82 vs. 72	Excluded	83 vs. 36	13.1 vs. 4.6	22.7 vs. 28.9	[13]
ENSURE	Chinese, EGFR mutated	Erlotinib vs. Cisplatin/Gemcitabine	110 vs. 107	Excluded	62.7 vs. 33.6	11.0 vs. 5.5	26.3 vs. 25.5	[14]
EURTAC	European, EGFR mutated	Erlotinib vs. platinum agent + Gemcitabine or Docetaxel	86 vs. 87	Excluded	58 vs. 15	9.7 vs. 5.2	19.3 vs. 19.5	[15]
LUX-Lung 3	Asian and Caucasian, EGFR mutated	Afatinib vs. Cisplatin/Pemetrexed	230 vs. 115	26 (11.3%) vs. 11 (9.6%)	56.1 vs. 22.6	11.1 vs. 6.9	25.8 vs. 24.5	[7,16]
LUX-Lung 6	Asian, EGFR mutated	Afatinib vs. Cisplatin/Gemcitabine	242 vs. 122	26 (10.7%) vs. 14 (11.5%)	66.9 vs. 23.0	11.0 vs. 5.6		[6,16]
LUX-Lung 7	Asian and Caucasian, EGFR mutated	Afatinib vs. Gefitinib	160 vs. 159	Excluded	70 vs. 56	11.0 vs. 10.9	27.9 vs. 24.5	[17]
ARCHER 1050	Asian and Caucasian, EGFR mutated	Dacomitinib vs. Gefitinib	227 vs. 225	Excluded	74.9 vs. 71.6	14.7 vs. 9.2	34.1 vs. 26.8	[18]
FLAURA	Asian and Caucasian, EGFR mutated	Osimertinib vs. Gefitinib or Erlotinib	279 vs. 277	Excluded	80 vs. 76	18.9 vs. 10.2	N.A.	[5]

* Patients with known EGFR-mutated status only. ** A post-hoc analysis of unanalyzed samples [9] revealed an EGFR mutation in 54 patients, which had previously been described as “EGFR mutation-unknown”, including 4.4% of exon 18 mutations and 6.6% double mutations. Legend: mos, months; ORR, overall response rate; PFS, progression-free survival; OS, overall survival.

**Table 2:** Activity of first-/second-generation EGFR TKIs in patients harboring exon 18 mutations, either alone or as complex mutations.

Study	*n*	Mutation(s) Included	EGFR TKI Used	ORR	DCR	PFS (mos) (95% CI)	OS (mos) (95% CI)
ERMETIC-IFCT network [19]	18	Single exon 18 mutations	G, E	7%	34%	3.0 (1–NE)	22.0 (1–44)
LUX-Lung -2, -3 & -6 pooled analysis [37]	18	Single G719X (8), complex G719X mutations (10)	A	77.8%	N.R.	13.8 (6.8–NE)	26.9 (16.4–NE)
Cheng C, 2015 [39]	5	Complex exon 18 mutations	G, E	80%	100%	N.R.	N.R.
Velcheti V, 2017 [40]	1	G721R	A	0%	0%	1.5	5.5
Wu JY, 2016 [41]	25	delE790_T710insD (5); complex E709X mutations (20)	G, E	50%	72.2%	6.2 (0.6–77.4)	29.3 (5.4–104.6)
Zhang Y, 2017 [42]	22	Single G719X (14), complex G719X mutations (8)	G, E, I	22.7%	90.9%	7.6 (4.9–10.4)	N.R.
Baek JH, 2015 [43]	7	Single exon 18 mutations	G, E	16.7%	100%	6.3 (0.0–14.7)	
Chen K, 2017 [44]	23	Single G719X (19); complex G719X mutations (4)	G, E, A, I	36.8% 0%	94.7% 100%	N.R.	N.R.
Frega S, 2017 [31]	9	Single G719X (4); Single E709X (3); complex mutations (2)	G, E, A	40%	40%	N.R.	N.R.
Keam B, 2014 [22]	4	Single G719A (2), complex G719A mutations (2)	G, E	33.3%	66.6%	N.R. (0.9–7.9)	N.R. (7.1–26.2)
Tu HY, 2017 [21]	16	Single G719X (12), complex G719X mutations (4)	G, E	50.0%	N.R.	11.6 (5.6–19.6)	25.2 (0–52.2)
Passaro A, 2019 [33]	42	Single exon 18 mutations	G, E, A	31.0%	69.0%	8.3 (4.8–11.7)	N.R.
BE-POSITIVE [20]	6	Single exon 18 mutations	G, E	0%	66.7%	8.38 (0.49–34.4)	17.0 (1.05–NE)
Xu J, 2016 [45]	14	Single G719X	G, E, I	42.9%	78.6%	5.98 (1.53–10.42)	19.81 (16.81–22.81)
Kobayashi S, 2013 [46]	3	Complex G719X mutations	E	100%	100%	N.R.	N.R.
Peng L, 2014 [47]	4	Complex G719A mutations (3), complex E709K mutations (1)	G	33.3%	100%	N.R.	N.R.
Wu JY, 2011 [36]	15	Single and complex G719X mutations	G, E	53.3%	N.R.	8.1	16.4
Chiu CH, 2015 [48]	78 9 10	Single G719X mutations G719X + L861Q G719X + S768I	G, E	36.8% 88.9% 50.0%	72.4% 100% 100%	6.3 N.R. N.R.	N.R. N.R. N.R.
NEJ-002 [12]	7	Single G719X mutations	G	14%	57%	N.R.	N.R.
Galli G, 2018 [49]	2	Single I706T mutation (1); Single G719A mutation (1)	A G	100% 0%	100% 0%	N.R.	N.R.
Sequist LV, 2010 [38]	4	Complex G719X mutations	N	75%	100%	12.1 (5.9–13.1)	N.R.

Legend: G, Gefitinib; E, Erlotinib; A, Afatinib; I, Icotinib; N, Neratinib, N.R. not reported; NE, not evaluable; ORR, overall response rate; PFS, progression-free survival; OS, overall survival.

**Table 3:** Activity of EGFR TKIs in patients with S768X mutation.

Study	*n*	Mutation(s) Included	EGFR TKI Used	ORR	DCR	PFS (mos) (95% CI)	OS (mos) (95% CI)
LUX-Lung -2, -3 & -6 pooled analysis [37]	8	Single S768I (1); complex S768I mutations (7)	A	100%	100%	14.7 (2.6–NE)	NE (3.4–NE)
Chiu CH, 2015 [48]	7 10	Single S768I; S768I + G719X	G, E	33.3% 50.0%	66.7% 100%	N.R. N.R.	N.R. N.R.
Levantakos K, 2016 [87]	4	Single S768I (1); complex S768I mutations (3)	E	25%	75%	N.R. (3.0–20)	N.R. (5.0–51.0)
Chen K, 2017 [44]	6	Single S768I (2); complex S768I mutations (4)	G, E, A, I	0% 0%	100% 50%	N.R.	N.R.
Zhang Y, 2017 [42]	11	Single S768I (4), complex S768I mutations (7)	G, E, I	27.3%	90.9%	8.0 (4.3–11.8)	N.R.
Frega S, 2017 [31]	1	Single S768R	G	0%	0%	N.R	N.R.
Peng L, 2014 [47]	1	S768I + L858R	G	0%	100%	6.0	6.5
Wu JY, 2011 [36]	4	S768I complex mutations	G, E	75%	75%	N.R.	N.R.

Legend: G, Gefitinib; E, Erlotinib; A, Afatinib; I, Icotinib; N, N.R. not reported; NE, not evaluable; ORR, overall response rate; PFS, progression-free survival; OS, overall survival.

**Table 4:** Activity of first-/second-generation EGFR in patients harboring L861Q single and compound mutations.

Study	*n*	Mutation(s) Included	EGFR TKI Used	ORR	DCR	PFS (mos) (95% CI)	OS (mos) (95% CI)
LUX-Lung -2, -3 & -6 pooled analysis [37]	16	Single L861Q (12), complex L861Q mutations (4)	A	56.3%	N.R.	8.2 (4.5–16.6)	17.1 (15.3–21.6)
NEJ-002 [12]	3	Single L861Q	G	33%	66%	N.R.	N.R.
Chiu CH, 2015 [48]	57 9	Single L861Q Complex L861Q	G, E	39.6% 88.9%	75.5% 100%	8.1 N.R.	N.R. N.R.
Wu JY, 2011 [36]	15	Single and complex L861Q	G, E	60%	N.R.	6.0	15.2
Zhang Y, 2017 [42]	5	Single L861Q (4), complex L861Q mutations (1)	G, E, I	0%	100%	5.7 (1.6–9.8)	N.R.
Chen K, 2017 [44]	16	Single L861 Q	G, E, A, I	31.3%	68.8%	N.R.	N.R.
Keam B, 2014 [22]	4	Single L861Q (3), complex L861Q mutations (1)	G, E	50%	75%	N.R. (0.8–7.9)	N.R. (0.9–26.2)
BE-POSITIVE [20]	5	Single L861Q	G, E	40%	60%	5.16 (1.58–22.3)	14.49 (5.55–NE)
Xu J, 2016 [45]	15	Single L861Q	G, E, I	46.7%	80.0%	8.90 (4.47–13.34)	21.98 (12.35–31.61)
Klughammer B, 2016 [63]	3	Single L861Q	E	33.3%	66.6%	N.R.	N.R.

Legend: G, Gefitinib; E, Erlotinib; A, Afatinib; I, Icotinib; N.R. not reported; NE, not evaluable; ORR, overall response rate; PFS, progression-free survival; OS, overall survival.
